# Association of body mass index and blood pressure variability with 10-year mortality and renal disease progression in type 2 diabetes

**DOI:** 10.1007/s00592-024-02250-z

**Published:** 2024-03-04

**Authors:** Stephen Fava, Sascha Reiff

**Affiliations:** 1 University of Malta Medical School, Msida, MSD 2090 Malta; 2 Department for Policy in Health, Valletta, Malta

**Keywords:** BMI variability, Blood pressure variability, Mortality, Renal disease progression, Type 2 diabetes

## Abstract

**Background:**

Variability in biological parameters may be associated with adverse outcomes. The aim of the study was to determine whether variability in body mass index (BMI) and blood pressure is associated with all-cause, cardiovascular mortality and cancer mortality or with renal disease progression in subjects with type 2 diabetes.

**Methods:**

The diabetes database was accessed, and all the information on patient visits (consultations) carried out in the study period (1 January 2008–31 December 2019) was extracted and linked to the laboratory database and the mortality register.

**Results:**

The total number of patients included in the study population was 26,261, of whom 54.4% were male. Median (interquartile range, IQR) age was 60.2 (51.8–68.3) years. The coefficient of variability of BMI was independently associated with increased all-cause and cardiovascular, but not cancer, mortality. Glycated haemoglobin (HbA_1c_) was associated with increased all-cause, cardiovascular, and cancer mortality as well as with renal progression. Variability in systolic blood pressure, diastolic blood pressure, and pulse pressure was associated with increased all-cause and cardiovascular mortality in bivariate, but not in multivariate, analyses.

**Conclusions:**

Variability in BMI was associated with increased all-cause and cardiovascular, but not cancer, mortality in a large real-world contemporary population. Our results also confirm the association of HbA_1c_ with increased all-cause, cardiovascular, and cancer mortality as well as with renal progression.

## Introduction

Variability in body mass index (BMI) has been associated with increased cardiovascular events and mortality in the general population [1–4], but this has not been confirmed by more recent studies [5–8]. Nam et al. [9] reported that in a Korean population with type 2 diabetes, body weight variability was associated with increased risks of MI, stroke, and all-cause mortality in patients with type 2 diabetes, but little data exist with respect to other racial groups. Recently, the Action for Health in Diabetes (Look AHEAD) trial in the US reported that fluctuations in BMI over a 4-year period were associated with adverse outcome in the control group but not in the intensive lifestyle intervention group [10]. Little data exist with regard to the relationship between fluctuations in BMI and cancer mortality or microvascular disease, but Chang et al. [11] recently reported that fluctuations in BMI were associated with increased risk of hepatocellular carcinoma in the general population, whilst a post hoc analysis of the ACCORD trial found that fluctuations in BMI in subjects with type 2 diabetes were associated with increased risk of renal events [12].

There are also little data on the effects of long-term blood pressure variability and outcomes in type 2 diabetes. Visit-to-visit variability in systolic blood pressure has been linked to increased all-cause mortality, coronary heart disease, stroke, and end-stage renal disease in the general population [13]. On the other hand, seasonal variability in blood pressure has been associated with better renal outcomes [14]. A retrospective study in China reported that visit-to-visit systolic blood pressure variability was a potential predictor for the development of cardiovascular and all-cause mortality in patients with type 2 diabetes [15].

The aim of the study was to determine whether variability in visit-to-visit BMI, systolic, diastolic blood pressure, and pulse pressure (as assessed by the coefficient of variation) is associated with (1) all-cause mortality; (2) cardiovascular mortality; (3) cancer mortality; (4) rise in albumin–creatinine ratio; and (5) progression of renal disease in subjects with type 2 diabetes.

## Methodology

The Malta national diabetes database was accessed, and all the information on patient visits (consultations) carried out in the study period (1 January 2008–31 December 2019) was extracted. Exclusion criteria included: all patients who passed away before the start of the study period; any test results and visit data outside of the study period; and patients younger than 18 years of age at the start of the study period.

The extracted information included patient unique identifier (the national ID number), gender, date of birth, visit dates, BMI, systolic blood pressure (SBP), diastolic blood pressure (DBP), and waist circumference. The national ID number was used to link the patients’ records to the National Mortality Registry—to extract the dates of death, and the underlying cause of death (using ICD10 coding). Causes of death were classified into cardiovascular (ICD-10 codes: I00-I09, I11, I13, I20-I51, I10, I12, I15, and I60-169) and cancer (ICD-10 codes: C00-C97, D00-D09, D10-D36, and D37-D48). It was also linked to the centralised Laboratory Information System so as to extract all the haemoglobin A_1c_ (HbA_1c_) and urinary albumin–creatinine ratio (ACR) results of tests which were done during the study period.

The date of birth was used to calculate the age of patients at the start of the study period. The SBP and DBP were used to calculate the pulse pressure (PP). The median and coefficient of variation for the BMI, SBP, DBP, and PP were calculated for every patient where the number of observations per patient during the study period was more than one. This was calculated as standard deviation divided by the mean of all observations during the study period.

ACR results were categorised into three groups, namely normoalbuminuria: ACR < 30 mg/g; microalbuminuria: 30–300 mg/g; and macroalbuminuria: ACR > 300 mg/g. Progession of kidney disease was defined as transition from normoalbuminuria at baseline to microalbuminuria or to macroalbuminuria, or transition of micro-albuminuria to macroalbuminuria. Those with macroalbuminuria at baseline were excluded from these analyses as they cannot progress. Therefore, non-progessors included subjects who are normoalbuminuric at baseline and remained normoalbuminuric and those who were microalbuminuric at baseline and remained microalbuminuric or regressed to normoalbuminuria.

### Statistical methods

Data tables were linked, and the data were analysed using R Studio running R version 4.2.3. Simple binary logistic regression was used for the continuous predictor variables and the Chi-square test for the factor predictor variables.

Multiple binary logistic regression was performed using backward elimination using a *p* value of < .05 as a cutoff to assess the relationship between the predictor and outcome variables: all-cause mortality, cardiovascular mortality, cancer mortality, and ACR progression which had a significant relationship in the bivariate analysis. Pulse pressure was not included in multivariate analyses since this would have violated one of the assumptions of multivariate analyses as the models also included systolic and diastolic blood pressure.

## Results

The total number of patients included in the study population was: 26,261, of whom 54.4% were male. The median (interquartile range, IQR) age at baseline was 60.2 (51.8–68.3) years. The median (IQR) HbA_1c_ was 7.10 (6.40–8.20) %, the median (IQR) BMI was 30.7 (27.3–34.6) whilst the median (IQR) waist circumference was 104 (96–112) cm. There was a median (IQR) of 11.0 (6.0–17.0) visits for every patient.

Tables 1, 2, and 3 show the results of bivariate analyses for all-cause, cardiovascular, and cancer mortality. Increasing age, higher HBA_1c_, ACR progression, higher systolic blood pressure, and higher pulse pressure were all associated with increased risk of all-cause and cardiovascular mortality. The coefficients of variation of BMI, systolic blood pressure, diastolic blood pressure, and pulse pressure were all associated with increased risk of all-cause and cardiovascular mortality. All-cause mortality was additionally associated with male gender and median ACR. Table 4 shows the results of multivariate analyses. Age was associated with increased all-cause mortality with an odds ratio (OR) (95% confidence interval, CI) of 1.14 (1.13–1.15); increased cardiovascular mortality with an OR (95% CI) of 1.11 (1.10–1.12); and increased cancer mortality with an OR (95% CI) of 1.06 (1.05–1.07) in multivariate analyses (*p* < .001 for all). HbA_1c_ was also associated with increased all-cause, cardiovascular, and cancer mortality with OR (95% CI) of 1.51 (1.43–159), 1.51 (1.41–163), and 1.14 (1.08–1.20), respectively (*p* < .001 for all). Male gender was independently associated with increased all-cause and cancer mortality.

**Table 1:** Patient characteristics by all-cause mortality

Characteristic	Survivors, *N* = 19,537	Fatalities, *N* = 6724	*p* value
Gender (male)	10,301/19,136 (54%)	3731/6677 (56%)	.004
Age at baseline	57.7 (49.19–63.81)	70.4 (62.26–76.86)	< .001
ACR^*^	14.33 (6.79–38.61)	37.75 (14.91–134.11)	< .001
ACR progression (yes)	638/6029 (11%)	190/1197 (16%)	< .001
HbA_1c_*	7.05 (6.40–8.10)	7.40 (6.60–8.55)	< .001
Waist circumference*	104 (96–112)	103 (95–112)	.12
Baseline BMI	30.9 (27.60–34.80)	29.6 (26.35–33.60)	< .001
BMI COV	0.02 (0.01–0.04)	0.03 (0.02–0.05)	< .001
SBP*	140.0 (130.0–150.0)	140.0 (129.0–154.0)^ǂ^	< .001
SBP COV	0.08 (0.05–0.11)	0.08 (0.05–0.13)	< .001
DBP*	80.0 (75.0–86.0)^ǂ^	80.0 (70.0–83.0)	< .001
DBP COV	0.07 (0.04–0.11)	0.08 (0.04–0.13)	< .001
PP*	58.0 (50.0–70.0)	60.0 (50.0–76.0)	< .001
PP COV	0.17 (0.10–0.25)	0.18 (0.11–0.28)	< .001

Data are median (IQR) for continuous variables and the ratio for factors

*Refers to median during study period. ^ǂ^Statistically higher category (higher sum of ranks)

*ACR* Albumin–Creatinine Ratio, *HbA*_*1c*_ Haemoglobin A_1c_, *BMI* Body Mass Index, *SBP* Systolic Blood Pressure, *DBP* Diastolic Blood Pressure, *PP* Pulse Pressure, *STD* Standard Deviation, *COV* Coefficient of Variation, and *IQR* Interquartile Range

**Table 2:** Patient characteristics by cardiovascular mortality

Characteristic	Survivors, N = 19,537	Fatalities, N = 6724	*p* value
Gender (male)	12,698/23,321 (54%)	1334/2492 (54%)	.39
Age at baseline	59.30 (50.91–66.67)	71.64 (63.58–77.67)	< .001
ACR*	16.00 (7.25–45.53)	44.36 (17.15–144.67)	.21
ACR progression (Yes)	759/6820 (11%)	69/406 (17%)	< .001
HbA_1c_*	7.10 (6.40–8.15)	7.40 (6.65–8.70)	< .001
Waist circumference*	104 (96–112)	103 (95–113)	.49
Baseline BMI	30.8 (27.40–34.70)	29.7 (26.4–33.9)	< .001
BMI COV	0.03 (0.01–0.04)	0.03 (0.02–0.05)	.02
SBP*	140.0 (130.0–151.0)	140.0 (128.5–155.1)^ǂ^	.003
SBP COV	0.08 (0.05–0.12)	0.09 (0.05–0.13)	< .001
DBP*	80.0 (74.0–85.5)^ǂ^	80.0 (70.0–83.0)	< .001
DBP COV	0.07 (0.04–0.11)	0.09 (0.04–0.14)	< .001
PP*	58.00 (50.00–70.00)	61.0 (50.0–79.0)	< .001
PP COV	0.17 (0.11–0.25)	0.19 (0.11–0.28)	< .001

Data are median (IQR) for continuous variables and the ratio for factors

*Refers to median during study period. ^ǂ^Statistically higher category (higher sum of ranks)

*ACR* Albumin–Creatinine Ratio, *HbA*_*1c*_ Haemoglobin A_1c_, *BMI* Body Mass Index, *SBP* Systolic Blood Pressure, *DBP* Diastolic Blood Pressure, *PP* Pulse Pressure, *STD* Standard Deviation, *COV* Coefficient of Variation, and *IQR* Interquartile Range

**Table 3:** Patient characteristics by cancer mortality

Characteristic	Survivors, N = 19,537	Fatalities, N = 6724	*p* value
Gender (male)	13,114/24,298 (54%)	918/1515 (61%)	< .001
Age at baseline	59.89 (51.28–67.87)	66.97 (60.06–73.81)	< .001
ACR*	16.83 (7.43–49.47)	26.00 (11.33–76.92)	.91
ACR progression (Yes)	789/6,966 (11%)	39/260 (15%)	.08
HbA_1c_*	7.10 (6.40–8.20)	7.20 (6.50–8.30)	< .001
Waist circumference*	104.0 (96.00–112.00)	102.0 (93.50–110.00)	< .001
Baseline BMI	30.8 (27.4–34.7)	29.30 (26.1–33.1)	< .001
BMI COV	0.03 (0.01–0.04)	0.03 (0.01–0.04)	.33
SBP*	140.0 (130.0–151.0)	139.5 (129.5–151.5)^ǂ^	.72
SBP COV	0.08 (0.05–0.12)	0.08 (0.05–0.12)	.47
DBP*	80.0 (74.0–85.0)^ǂ^	80.0 (71.0–84.0)	< .001
DBP COV	0.07 (0.04–0.12)	0.07 (0.04–0.11)	.75
PP*	59.0 (50.0–70.0)	60.0 (50.0–73.0)	.009
PP COV	0.17 (0.10–0.25)	0.17 (0.11–0.28)	.09

Data are median (IQR) for continuous variables and the ratio for factors

*Refers to median during study period. ^ǂ^Statistically higher category (higher sum of ranks)

*ACR* Albumin–Creatinine Ratio, *HbA*_*1c*_ Haemoglobin A_1c_, *BMI* Body Mass Index, *SBP* Systolic Blood Pressure, *DBP* Diastolic Blood Pressure, *PP* Pulse Pressure, *STD* Standard Deviation, *COV* Coefficient of Variation, and *IQR* Interquartile Range

**Table 4:** Independent predictors of all-cause, cardiovascular, and cancer mortality in a binary logistic model

Characteristic	Odds ratio	95% confidence intervals	*p* value
*All-cause mortality*			
Gender—male	1.61	1.42, 1.84	< .001
Age at baseline	1.14	1.13, 1.15	< .001
HbA_1c_*	1.51	1.43, 1.59	< .001
BMI COV	14.6	3.91, 52.7	< .001
*Cardiovascular mortality*			
Age at baseline	1.11	1.10, 1.12	< .001
HbA_1c_*	1.51	1.41, 1.63	< .001
BMI COV	7.37	1.10, 39.9	.028
*Cancer mortality*			
Gender—male	1.45	1.26, 1.68	< .001
Age at baseline	1.06	1.05, 1.07	< .001
HbA_1c_*	1.14	1.08, 1.20	< .001
Baseline BMI	0.99	0.97, 1.00	.048

*Refers to median during study period

*HbA1c* Haemoglobin A_1c_, *BMI* Body Mass Index, and *COV* Coefficient of Variation

BMI variability, as assessed by the coefficient of variation, was associated with increased all-cause and cardiovascular mortality with OR (95% CI) of 14.6 (3.9–5.7, *p* < .001) and 7.37 (1.10–39.9, *p* = .028), respectively.

Only age, HbA_1c_, ACR, and pulse pressure were associated with ACR progression in bivariate analysis (Table 5). Age and HbA_1c_ remained significant in multivariate analyses (Table 6).

**Table 5:** Patient characteristics by ACR progression

Characteristic	No, N = 6398	Yes, N = 828	*p* value
Gender (male)	3752/6330 (59%)	479/818 (59%)	.72
Age at baseline	59.74 (52.17–65.64)	61.99 (55.07–68.30)	< .001
ACR*	16.50 (8.18–43.07)	60.52 (40.84–137.70)	< .001
HbA_1c_*	7.35 (6.60–8.40)	7.55 (6.80–8.60)	< .001
Waist circumference*	104.00 (96.00–112.00)	104.25 (97.00–112.00)	.22
Baseline BMI	30.90 (27.70–34.70)	30.60 (27.60–34.40)	.15
BMI COV	0.03 (0.01–0.04)	0.03 (0.02–0.05)	.39
SBP*	140.0 (130.00–152.5)	140.0 (130.0–155.0)	.13
SBP STD	10.0 (5.44–14.50)	10.00 (6.00–14.77)	.74
SBP COV	0.08 (0.05–0.12)	0.08 (0.05–0.12)	.70
DBP*	80.0 (74.0–86.5)	80.0 (72.0–85.0)	.03
DBP STD	5.01 (3.00–7.80)	5.50 (3.09–8.23)	.21
DBP COV	0.08 (0.05–0.12)	0.08 (0.05–0.12)	.13
PP*	60.0 (50.0–71.5)	61.0 (50.0–75.0)	.004
PP STD	8.76 (5.00–12.95)	8.96 (5.28–12.92)	.78
PP COV	0.17 (0.11–0.25)	0.17 (0.11–0.24)	.26

Data are median (IQR) for continuous variables and the ratio for factors

*Refers to median during study period

*ACR* Albumin–Creatinine Ratio, *HbA*_*1c*_ Haemoglobin A_1c_, *BMI* Body Mass Index, *SBP* Systolic Blood Pressure, *DBP* Diastolic Blood Pressure, *PP* Pulse Pressure, *STD* Standard Deviation, *COV* Coefficient of Variation, and *IQR* Interquartile Range

**Table 6:** Independent predictors of albumin–creatinine ratio progression in a binary logistic model

Characteristic	Odds ratio	95% confidence intervals	*p* value
Age at baseline	1.03	1.02, 1.03	< .001
HbA_1c_*	1.17	1.11, 1.24	< .001

*Refers to median during study period

*HbA1c* Haemoglobin A_1c_

## Discussion

We found that variability of BMI, as assessed by the coefficient of variation, was associated with increased all-cause and cardiovascular, but not cancer, mortality in a population with Caucasian type 2 diabetes. This confirms what has been previously reported in a Korean population [9]. Our 10-year follow-up is the longest to report an association between fluctuation in BMI and cardiovascular outcome, whilst there are little data with respect to cancer mortality. Our results on fluctuating BMI are important because weight cycling is thought to be common, even in normal weight individuals [16] as a result of repeated attempts to lose weight and difficulty to maintain weight loss. Weight cycling is also associated with impaired psychological health [17]. There is evidence that weight loss beyond an individual’s set point is hindered by hormonal and metabolic changes [18]. It may, therefore, be important to set realistic targets for weight loss as this may be healthier than weight cycling, especially in individuals who have difficulty in maintaining weight loss.

Fluctuating BMI is due to repeated cycles of lipolysis and lipogenesis. Lipolysis results in increased circulating free fatty acids, which are known to be pro-inflammatory and to induce insulin resistance [19–22] and oxidative stress [23]. In fact, BMI cycling has been associated with hyperinsulinaemia, the metabolic syndrome, and higher pro-inflammatory markers [24–26]. The ‘repeated overshoot’ theory postulates that repeated overshoot of some cardiovascular factors during the weight regain phase of weight cycling may contribute to overall morbidity and mortality [16]. Weight cycling may also lead to preferential visceral fat accumulation [27], which is thought to be more detrimental. There is also evidence that weight gain following weight loss results in rapid expansion and hyperplasia of adipocytes [27], leading to an adverse adipokine profile. Variability in BMI was not independently associated with renal progression in our study, suggesting that the above mechanisms are less important in diabetic kidney disease.

Our results also confirm the association of HbA_1c_ with increased all-cause, cardiovascular, and cancer mortality as well as with renal progression in a large real-world contemporary population. This is important since to date there are no convincing data that intensive glycaemic control improves survival. For example, a Cochrane review of 28 trials with a total 34,912 T2D participants found that intensive glycaemic control did not show significant differences for all-cause mortality and cardiovascular mortality control compared with conventional glycaemic control [28]. The reason for the failure of randomised controlled to find a beneficial effect of tight glycaemic control may be due to the short duration of many of them. In fact, the UK Prospective Study marginally missed statistical significance at 10 years [29], but demonstrated statistically lower all-cause mortality after a further 10-year observation period [30]. Most other trials were of shorter duration. Another reason could be the hypoglycaemia risk associated with some of the anti-hyperglcaemic agents used in many of these older studies.

We also report an association of HbA_1c_ with cancer mortality. There are currently little data in the literature on the long-term effect of glycaemic control on cancer mortality, although a few recent studies have shown improved survival with better glycaemic control after diagnosis of some specific cancer types [31–33]. If confirmed by other authors, our findings may have important public health implications.

Our data show an association of variability in systolic blood pressure, diastolic blood pressure, and pulse pressure with increased all-cause and cardiovascular mortality in bivariate analyses. This is similar to the findings of earlier studies [34, 35] and to the results of post hoc pooled analysis of the Canagliflozin Cardiovascular Assessment Study (CANVAS) and Canagliflozin and Renal Events in Diabetes with Established Nephropathy Clinical Evaluation (CREDENCE) studies [36]. However, the relationship lost statistical significant in multivariate analysis in the present study. It should be noted that, unlike the previous studies, we also adjusted for variability in BMI in our multivariate analyses. This suggests that the previously reported association between variability in blood pressure and adverse cardiovascular outcome and all-cause mortality might be mediated through variability in BMI, given the known relationship between blood pressure and BMI [37]. This merits further study. Our data also show relationship between blood pressure variability and renal progression. However, this again lost statistical significance in multivariate analysis, suggesting that the association is mediated through the confounding effect of other parameters such as advancing age, which is known to be associated with higher blood pressure variability [38]. Our data are consistent with the pooled analysis of the CANVAS and CREDENCE studies referred to above, which also found that SBP variability was not associated with kidney outcomes [36].

In our study, baseline BMI was lower in non-survivors. We studied baseline rather than the mean BMI during the study period in order to minimise the possibility of reverse causality, namely that cardiac disease or malignancy leads to weight loss. The association of a higher BMI with lower cardiovascular [39–42] and cancer [43–45] mortality has been previously reported and is often referred to as the obesity paradox. The reasons for this are unclear, given the known association of adiposity with adverse outcomes [46]. However, the association lost statistical significance in multivariate analyses for all-cause and cardiovascular mortality in the present study. This suggests that the association between lower BMI and improved all-cause and cardiovascular survival is mediated by other factors such as decreasing BMI with advancing age. Reduction in BMI with advancing age is often due to due to loss of muscle, rather than fat, mass [47]. Loss of muscle mass can lead to insulin resistance [48], which is known to be associated with increased all-cause, cardiovascular disease [49–51] and cancer [51–53] mortality. Decreasing BMI may also be a marker of frailty, which is also known to be associated to increased mortality [54]. This is supported by the fact that we did not find an association of waist circumference with all-cause or cardiovascular mortality. The association remained significant with regard to cancer mortality in multivariate analysis. Possible explanations include weight loss as a result of occult malignancy or the presence of residual confounders such glycaemic control before the study period.

### Strengths and limitations

One strength of our study is that we could access and link robust databases through the use of a unique identifier. These included the national diabetes electronic health record, the national health system’s laboratory information system, and the national mortality register. Other strengths include the large number of patients studied and the long follow-up period. Furthermore, the fact these databases included patients being cared for in primary care, secondary care, or both means that our results are more generalisable to the type 2 diabetes population. Nonetheless, our results need to be confirmed by other authors studying other populations and racial groups. Another limitation is that we do not have data on lifestyle factors such as smoking, diet, or exercise. Our study was not designed to study the relationship between BMI and blood pressure variability. Therefore, the possibility raised by our data that the association between blood pressure variability and adverse cardiovascular outcome and all-cause mortality may be mediated through variability in BMI needs confirmation in future appropriately designed studies.

## Conclusions

Variability of BMI, as assessed by the coefficient of variation, was associated with increased all-cause and cardiovascular, but not cancer, mortality in a large real-world contemporary population. Our results also confirm the association of HbA_1c_ with increased all-cause, cardiovascular, and cancer mortality as well as with renal progression. Furthermore, our results suggest that the previously reported association between variability in blood pressure and adverse cardiovascular outcome and all-cause mortality might be mediated through variability in BMI. Furthermore, our data provide further insights into the obesity paradox.
